# Nutritional Comparison of White and Red *Coccinia Abyssinica* (Lam.) Cong. Accessions: An Under-Utilised Edible Tuber of the Ethiopian Highlands

**DOI:** 10.3390/foods6080071

**Published:** 2017-08-22

**Authors:** Aditya Parmar, Bilatu Agza Gebre, Addisu Legesse, Yoseph Demelash, Kirsten Fladung, Oliver Hensel

**Affiliations:** 1 Department of Agricultural and Biosystems Engineering, University of Kassel, D-37213 Witzenhausen, Germany; agrartechnik@uni-kassel.de; 2 Ethiopian Institute of Agricultural Research, Head Office, P.O.Box 2003 Addis Ababa, Ethiopia; bilatuagza@yahoo.com; 3 Department of Food Science and Nutrition, Ethiopian Public Health Institute, Gulele Sub City, Addis Ababa, Ethiopia; addislegesse@yahoo.com (A.L.); yose.deml@gmail.com (Y.D.); 4 Institute of Applied Plant Nutrition, Georg-August University Goettingen, D-37075 Göttingen, Germany; kirsten.fladung@agr.uni-goettingen.de

**Keywords:** anchote, *Coccinia abyssinia*, Ethiopia, edible root and tubers, under-utilised crops

## Abstract

Anchote (*Coccinia abyssinica*) is an indigenous tuber crop of the Ethiopian Highlands. It is popular in the western Oromia Region of the country. Apart from food, the crop is also used in traditional medicine. Anchote tubers possess two variations in its tissue colour, red and white. In this study, a small market survey and a nutritional comparison of red and white anchote were conducted. White tissue anchote seems to be more popular, due to its soft texture and ease of cooking. However, the red variant was considered for flour making (by dehydration), for use in porridge and soups for various medicinal and supplementary food applications. Red anchote tubers contained significantly higher protein content (16.85 mg/100 g dry matter basis) than the white variant. However, apart from the marginally higher protein content compared to other tropical root and tuber crops, anchote seems to remain a primary source of carbohydrates. In macro minerals, white anchote proves to be a more important source of Ca with 81 mg/100 g edible portion; however, on dry matter basis, the content was similar to the red variant (316 and 309 mg/100 g dry matter, white and red respectively). Further research on vitamin content (especially vitamin A in the red variant) would be useful to understand the full nutrition potential of the crop.

## 1. Introduction

**Figure 1:**
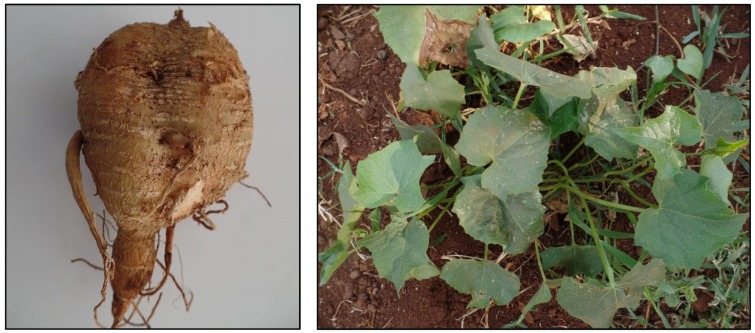
Anchote (*Coccinia abyssinica*) tuber and plant. (Pictures by Aditya Parmar).

Ethiopia is a biodiversity hotspot, thanks to its highly diversified climate and edaphic conditions. More than 6000 flora species residing in 23 different vegetation types have been recognised in the country, of which 12% of the species are indigenous [1]. The indigeneity is particularly high on the highlands of southwestern and western regions. Coffee (*Coffea arabica*), safflower (*Carthamus tinctorius*), tef (*Eragrostis tef*), noug (*Guizotia abyssinica*), and enset (*Ensete ventricosum*) are some of the examples of crops which have originated in Ethiopia. Apart from cereals and pulses, Ethiopian agroecosystems are highly suitable for the production of high-quality roots and tubers. Getahun [2] listed about 30 edible starchy root and tuber crops from Ethiopia. *Coccinia abyssinica* (Lam.) Cogn., commonly known by its vernacular name anchote (Figure 1), is one of these root and tuber crops. Cultivation of anchote is particularly widespread in the western and southwestern regions of the country, at varying elevations of 1300–2800 m with an annual rainfall of 762–1016 mm [3,4].

Anchote is the only plant in the *Cucurbitaceae* family which is known to produce edible starchy tubers [5,6]. Culturally, in Oromia region, women are responsible for the breeding, cultivation, harvesting, and processing of the crop [7,8]. Anchote is not only grown for home consumption, but also for sale; apart from tubers as food, anchote seeds and seedlings for propagation are some of the items which are marketed. The simplest preparation of anchote is boiling the harvested tubers and peeling them, before eating with some salt and ground pepper. Other more complex preparations involve the addition of many spices and butter, made into a paste and eaten alone or with local bread [3,9].

Anchote produces one or two tubers per plant on average, and stems are vines which can grow up to 2 m in height [7]. Regarding genetic diversity, the crop has been found to be highly diversified in its characteristic (tuber length, diameter, and yield per plant) [4,5,10]. Based on the underlying (tissue) colours, two anchote accessions are well known among the farmers, locally called (in the Oromo language) as *dimma* (red or deep orange) and *addi* (white) [3,11]. From the outside, both types look similar, and the only way to discover the tissue colour (red or white) is to remove the corky skin. Despite limited research on anchote, certain recent studies have presented its nutritional and anti-nutritional factors [6,12,13]. However, in all these publications there is no mention of the tissue colour. Anecdotal evidence suggests that red tissue anchote is more valued among the local population for its medicinal properties. In the Oromo region (especially in the Wellega zone) soups and juice made of red anchote are frequently recommended to individuals suffering from fractures and displaced joints, as well as to lactating mothers [2,3,9].

The objective of this paper was to make a comparison of the macro-nutritional composition for red and white anchote accessions, to verify the anecdotal hypothesis that red accessions are more nutritious, and to present a potential of the crop as a functional and supplementary food. Apart from that, a short survey among various value chain actors was conducted to understand the involved actors, the cooking process, what all kind of anchote dishes are available in the market and what the various medicinal uses of the tuberous crop are.

## 2. Materials and Methods

### 2.1. Survey, Collection, and Sample Preparation

**Figure 2:**
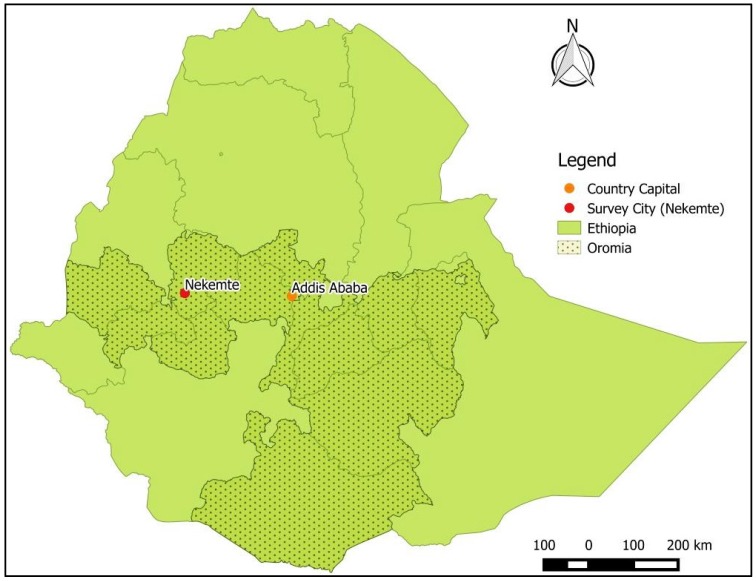
Study site (Nekemte Coordinates: 9.0893° N, 36.5554° E; Elevation 2,088 m).

A short survey of the fresh anchote market, farmers (visiting to sell their products at the market), and local restaurants which serve various anchote-based dishes was conducted in Nekemte, Oromia, Ethiopia (the location is presented in Figure 2). In total, about three farmers, seven anchote chefs, and two retailers (who sell processed anchote flour for medicinal purposes) were interviewed using a semi-structured questionnaire.

**Figure 3:**
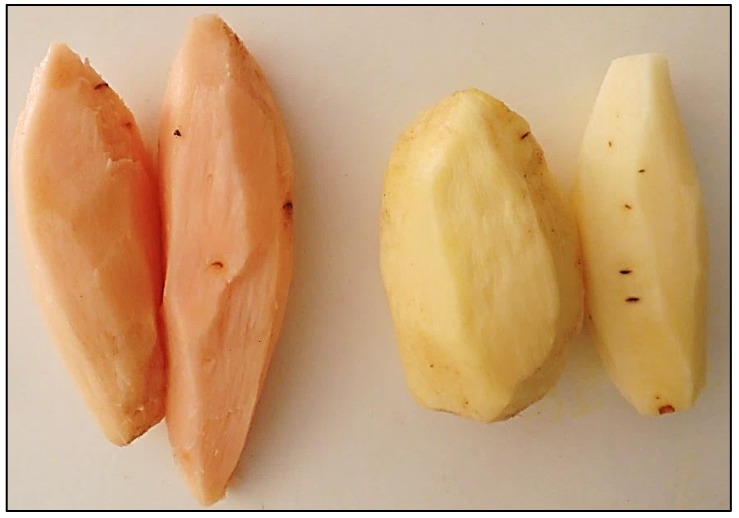
Red (left) and white (right) colour parenchyma tissue of anchote after peeling. (Picture by Aditya Parmar).

Fifteen tubers (five tubers per replica) of each anchote tuber accession (red and white) were collected randomly from a one-day-old harvest at a local vegetable market in Nekemte and a neighbouring town (Negassa). Red and white parenchyma tissues of anchote are illustrated in Figure 3. It is hard to distinguish red and white tissue anchote from its external appearance; hence a small portion of the corky skin was removed to identify underlying red and white parenchyma tissue visually. However, in most cases, the tubers which have been transported to retail market have skinning injuries and small bruises due to improper packaging and handling which makes it easier to select the two colour accessions. Tubers were packed in paper bags and transported to the Ethiopian Institute of Agricultural Research (EIAR) and the Ethiopian Public Health Institute (EPHI), Addis Ababa, for further preparation and analysis. To some degree, International Potato Center (CIP) guidelines [14,15] for sample collection and preparation were followed, which involved first washing the tubers (with tap water) to remove all soil residues and then rinsing with distilled before drying the unpeeled tubers with a clean paper towel. Tubers were peeled with a high-grade stainless steel handheld peeler, and were again washed with distilled water and dried using a paper towel before they were sliced using a high-grade stainless steel knife. Due to the unavailability of a freeze dryer, low-temperature oven drying (at 40 °C for 72 h) was employed to obtain dehydrated samples with a moisture content below ~10%. The dried samples were milled using a stainless steel mill and packed and stored (at room temperature) into sealed plastic bags for proximate and mineral analysis.

### 2.2. Proximate Analysis

Proximate composition (in dry matter basis) of the oven-dried samples was conducted following the AOAC (Association of Official Analytical Chemists) [16] standard procedures at EIAR and EPHI. Later on, the data was recalculated on the 100 g raw fresh basis to compare with other common tropical root and tuber crops. The micro-Kjeldahl procedure was used for the crude protein (N × 6.25) analysis following method 954.01, AOAC (2010). The moisture content (MC) was determined by the hot air oven method as described by 925.10, AOAC (2010). Fat content was determined using the Soxhlet extraction with hexane according to method number 2003.06, AOAC (2010). The method 923.03, AOAC (2010) was used to determine the total ash content. Method number 962.09, AOAC (2010), was used for crude fibre determination. Carbohydrate content was calculated by difference as shown: Total carbohydrate by difference = 100 – (water, protein, total lipid, ash in g/100 g). Standard energy conversion factors (protein 4 kcal/g, fat 9 kcal/g, and carbohydrate 4 kcal/g) were used to estimate energy (in kcal).

### 2.3. Mineral Analysis

Macro (Ca, Mg, P, K, Na, S) and micro (Cu, Co, Fe, Mn, Se, Zn) minerals analysis of red and white dried anchote flour were conducted at the Institute of Applied Plant Nutrition, Georg-August University Goettingen, Germany. The standard method, EPA (United States Environmental Protection Agency) 6010Aof Inductively Coupled Plasma-Atomic Emission Spectrometry (ICP-AES) (VARIAN VISTA RL CCD Simultaneous ICP-AES Spectrometer) was followed. Microwave digestion (100 g dry plant material using conc. HNO_3_ and 30% H_2_O_2_) with wet ashing under pressure (for 75 min, 200 °C and 15 bar) was used to prepare samples prior to ICP-AES analysis. Mineral content was recorded in mg/kg of flour and later converted into mg/100 g of dry matter or fresh weight basis.

### 2.4. Statistics

Statistical significance was determined by using a Microsoft Excel *t-Test* of the paired two sample for means.

## 3. Results and Discussions

### 3.1. Survey

**Figure 4:**
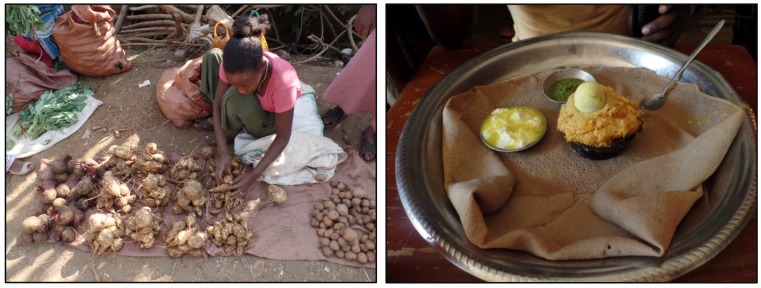
Display of anchote tuber with other crops at a local vegetable market in Nekemte (left), and a typical anchote dish (namely *special anchote*, with butter, Jogurt, and Injera (local bread)) (right). (Picture by Aditya Parmar).

Anchote is a high-value crop; the retail price (at Nekemte local vegetable market) of the tuber was 4–5 times greater than other tropical root and tuber crops such as taro and sweet potato. Supply of the anchote remains continuous in all seasons (in dry season plantation, irrigation is used) due to its high demand from restaurants. Anchote chefs and retailers stated that the white tissue anchote is preferred over red tissue due to its soft texture and ease of cooking. Locals believe that the red anchote has higher medicinal values. In general, anchote tubers are hard and take a longer time to cook, when compared to other tropical starchy tubers such as cassava (*Manihot esculenta* Crantz) and sweet potato (*Ipomoea batatas* (L.) Lam.). One of the respondents (a well-known anchote chef in Nekemte and owner of Irsha Seble Cafe and Restaurant, Age Mengistu) mentioned that a significant amount of fuel wood is required to cook anchote dishes and cooking can take as long as 3 h. In Ethiopia, anchote is associated with traditions of the Oromo tribe. Various anchote dishes are prepared for ceremonies (wedding, birthdays) and festivals (Meskel (traditional Ethiopian festival), New Year) in Oromia Region (land of the Oromo people). A typical retail display of anchote tuber and the most common preparation are presented in Figure 4.

In total, there may be more than 27 different types of dishes which can be prepared from the anchote tuber. However, there are four broad categories, which are as follows: soups, boiled anchote, dehydrated anchote (flour), and anchote dishes (main courses). Soups and dehydrated anchote (prepared in the form of porridge with milk and salt) is mostly used as food for children, the elderly, and lactating mothers. The soups and dehydrated anchote flour are more commonly prepared from red tissue anchote due to its believed higher medicinal properties. In local traditional medicines, the anchote in the form of soups and porridges (red tissue tuber) is considered for people suffering from bone related problems (fractures, displaced joints), as well as pregnant women and lactating mothers.

### 3.2. Proximate Analysis

The proximate analysis suggests that anchote tubers have a marginally higher protein content than other common root and tuber crops in the region (such as cassava and sweet potato). Red anchote contained a significantly higher level of crude protein (*p* value = 0.041), with an average of 16.85 and 9.72 mg/100 g (on a dry matter basis) forred and white accessions respectively. Anecdotal evidence during the market survey suggested that anchote chefs and processors try to label the crop as a rich source of protein. Hora [3] also mentioned that anchote could contribute a significant amount of protein in rural diets. However, the protein content remains low (Table 1) according to dietary reference intakes (DRIs), [17], which recommends an adequate daily intake of protein ranging from 13 g/day for children (1–8 years) and 71 g/day for pregnant and lactating women. Limited information is available on the amino acid composition and protein quality of the anchote tuber. Only recently (in December 2016), Ayalew et al. [18] provided some details on the amino acid profile of anchote tuber protein. The essential amino acids for the tuber varied from 32.98–41.63% with an average of 37.22% [18]. The result from this study further added that arginine was the highest and methionine was of the lowest concentration with 6.5–9.5 and 0.3–0.4 g/100 g protein, respectively [18]. However, Ayalew et al. [18] also does not distinguish the tuber on based on its internal tissue colour, so these results can be assumed to be applicable to both accessions.

The proximate composition suggests that on a macro nutrient level, anchote remains a source of carbohydrates like other starchy root and tuber crops. The white tissue anchote contains 36.67% higher kcal in comparison to red anchote, whereas lipids and dietary fibres do not represent any difference among two accessions. The higher energy values for the white accession could be due to the presence of a higher level of starch content. Moreover, the dry matter content of red anchote was found to be significantly lower (*p* value = 0.0002) in comparison to the white tissue accession. Lower dry matter content may result in a lower shelf-life of the red accession.

The results presented in Table 1 more or less correspond to the values provided by Hora [3], who mentioned water, protein, fat, fibre, and carbohydrate content of 74, 4.2, 0.12, 1.73, and 21.1 g/ 100 g fresh weight of an edible portion, respectively. In a recent study by Aga and Badada [13], the protein and carbohydrate content of the whole anchote was reported as 3.0 and 22.5 g/100 g per edible portion, which again corresponds to the level of protein in the two types of anchote analysed in this study. However, Hora [3] and Aga and Badada [13] did not specify the tissue colour of the anchote used for analysis; hence, it is not possible to provide a comparison of red and white accessions.

### 3.3. Mineral Analysis

Ca content is perhaps the most highlighted mineral constituent of the anchote. The richness in Ca is mentioned in many nutritional analyses which were conducted on the crop [3,6,12,13]. These previous studies mentioned a Ca content (mg/100 g of edible portion) ranging from 119 mg to 344 mg; however, again the type of anchote (red or white) was not mentioned. In the current analysis on dry matter basis, the Ca content of white and red tissue anchote was 316 and 309 mg/100 g dry matter. However, when the calculations were made for raw, peeled, unprepared anchote (Table 2), the white anchote seemed to be a richer source of Ca (because of the higher dry matter presence). The Ca content of anchote is also associated to its hearsay medicinal properties to provide speedy recovery in bone fractures and displaced joints.

The Ca content of anchote is five to three times higher than that of cassava and sweet potato, which does make it an important source of plant-based Ca for the local population. A 100-g serving of anchote may provide about 10% of the daily requirement of Ca [17]. The Mg content of red and white tissue anchote is about 50 mg/100 g per edible portion, which is more than one third of the daily requirement for children aged 4–8 years, and is two times higher than that of cassava and sweet potato. Red anchote had considerably higher P (516 mg/100 g dry matter) than white anchote (313 mg/100 g dry matter basis). In a 100-g portion of raw, unprepared anchote, the P content is two to three times greater than that of sweet potato and cassava. In a 100-g sample of dry matter, red anchote contains about 167 mg of S, which is considerably higher than that in the white accession (121 mg/100 g dry matter). In micro minerals, the Se in white tissue anchote seems to attract the most attention as it may provide close to 76% of the daily required amount of 55 µg (DRIs, 2011) for an adult male. Apart from that, anchote is a moderate supplier of micro minerals such as Cu and Zn. In a dry matter sample, the Fe content of red and white anchote tubers was 4.69 and 3.80 mg /100 g, respectively. In comparison to cassava, anchote presents a three times higher Fe content. However, at an overall nutritional level (the DRI of Fe for children 4–8 years of age is 10 mg/day), the concentration remains low.

## 4. Conclusions

Anchote is a popular tuberous crop in the western Oromia region (especially in the Wellega zone). The crop holds a good regard in the area due to it close traditional ties with the Oromo people. In the present study, it was found that white tissue anchote was a regular daily food, served in various cooked forms in restaurants and homes, whereas red tissue anchote was considered appropriate for food supplementation for lactating mothers, children, and the elderly. Perhaps this may be due to the presence of a higher protein content in the red variant. In comparison to other tropical root and tuber crops (cassava and sweet potato), anchote tubers are richer in protein as well as various minerals such as Ca, Mg, P, Fe, and S.

Further studies regarding the carotenoids analysis of the red tissue anchote can provide more evidence about its applicability towards the alleviation of vitamin A deficiency, especially among children and pregnant women. More research and development in improving the anchote varieties to obtain higher nutritional content are required. Awareness about the crop among other regions in Ethiopia is still limited, and promotion of the anchote as a complementary food can improve the under-utilised status of this Ethiopian Highland tuber crop.

## Figures and Tables

**Table 1:** Proximate composition value per 100 g (raw, peeled, unprepared, edible portion) of red and white *Coccinia abyssinica* and a comparison with cassava and sweet potato.

Proximates	Units	White Anchote (*C. Abyssinica*)	Red Anchote (*C. Abyssinica*)	Cassava (*M. Esculenta*) ^1^	Sweet Potato (*I. Batatas* ) ^2^
Water (fresh weight)	g	71.47	78.76	59.68	77.28
Energy	kcal	111.77	81.78	160	86
Protein	g	2.77	3.58	1.36	1.57
Total lipid (fat)	g	0.41	0.26	0.28	0.05
Total carbohydrates (by difference) ^3^	g	24.25	16.27	38.06	20.12
Crude fibre	g	1.26	0.95	1.8 ^4^	3.0 ^4^
Total Ash	g	1.1	1.12	-	-

^1,2^ United States Department of Agriculture (USDA), Agricultural Research Service, National Nutrient Database for Standard Reference Release 28; ^3^ Total Carbohydrate = 100 − (Water + Protein + Fat + Ash); ^4^ Total dietary fibre.

**Table 2:** Minerals available in 100 g (raw, peeled, unprepared, edible portion) of red and white *Coccinia abyssinica*, and comparison with cassava and sweet potato.

Minerals	Units	White Anchote (*C. Abyssinica*)	Red Anchote (*C. Abyssinica*)	Cassava (*M. Esculenta*) ^1^	Sweet Potato (*I. Batatas*) ^2^
*Macro*					
Ca	mg	81.16	59.13	16	30
Mg	mg	50.30	50.33	21	25
P	mg	80.41	98.72	27	47
K	mg	315.83	313.01	271	337
Na	mg	5.763	5.87	14	55
S	mg	31.27	32.06	-	-
*Micro*					
Cu	µg	124	151	-	-
Co	µg	n.n.	2	-	-
Fe	mg	0.98	0.90	0.27	0.61
Mn	mg	0.29	0.28	-	-
Se	µg	42.30	7.0	-	-
Zn	mg	0.58	0.58	0.34	0.3

^1,2^ United States Department of Agriculture (USDA), Agricultural Research Service, National Nutrient Database for Standard Reference Release 28.
